# Somatic *GNAS* mutations in acromegaly: prevalence, clinical features and gender differences

**DOI:** 10.1530/EC-24-0266

**Published:** 2024-12-20

**Authors:** Yamei Yang, Yong Yao, Kan Deng, Bin Xing, Wei Lian, Hui You, Feng Feng, Xin Lian, Xinxin Mao, Fengying Gong, Linjie Wang, Meiping Chen, Xiaoan Ke, Hui Miao, Lian Duan, Huijuan Zhu

**Affiliations:** ^1^ Department of Ultrasound, State Key Laboratory of Complex Severe and Rare Diseases, Peking Union Medical College Hospital, Chinese Academy of Medical Sciences and Peking Union Medical College, Beijing, China; ^2^ Department of Neurosurgery, Peking Union Medical College Hospital, Chinese Academy of Medical Science and Peking Union Medical College, Beijing, China; ^3^ Department of Radiology, Peking Union Medical College Hospital, Chinese Academy of Medical Science and Peking Union Medical College, Beijing, China; ^4^ Department of Radiation Oncology, Peking Union Medical College Hospital, Chinese Academy of Medical Science and Peking Union Medical College, Beijing, China; ^5^ Department of Pathology, Peking Union Medical College Hospital, Chinese Academy of Medical Science and Peking Union Medical College, Beijing, China; ^6^ Department of Endocrinology, State Key Laboratory of Complex Severe and Rare Diseases, The Translational Medicine Center, Key Laboratory of Endocrinology of National Health Commission, Peking Union Medical College Hospital, Chinese Academy of Medical Science and Peking Union Medical College, Beijing, China

**Keywords:** acromegaly, *GNAS* mutations, tumor size, GH, gender

## Abstract

**Background:**

Somatic *GNAS* mutations are acknowledged as a significant etiological factor for acromegaly. However, the relationship between *GNAS* mutation status, clinical characteristics and gender has not been adequately investigated. This study aims to address these gaps by examining *GNAS* mutations and delineating the detailed clinical profile of affected patients within a Chinese acromegaly cohort.

**Methods:**

Our study encompassed 97 individuals newly diagnosed with acromegaly who underwent surgical treatment between May 2015 and January 2022. We obtained DNA from frozen pituitary adenomas to screen for *GNAS* hotspot mutations and assessed the associated clinical characteristics.

**Results:**

In our cohort, 44.3% (43/97) of patients exhibited somatic *GNAS* mutations. Patients with mutations were predominantly male (58.1 vs 33.3%, *P* = 0.015), experienced longer diagnosis delays (72.0 (48.0, 120.0) vs 36.0 (21.0, 75.0) months, *P* = 0.002), had smaller maximum tumor diameters (1.75 ± 0.83 vs 2.23 ± 0.89 cm, *P* = 0.008) and demonstrated higher rates of growth hormone (GH) secretion per unit tumor volume (18.93 (9.67, 30.12) vs 10.91 (2.80, 20.40) ng/mL cm^−3^, *P* = 0.005). Regarding gender-specific differences, *GNAS* mutations in male patients were linked to significantly higher baseline GH levels (24.40 (14.40, 36.30) vs 10.55 (5.25, 16.95) ng/mL, *P* = 0.002), while female patients with mutations had notably smaller tumor sizes (1.55 ± 0.55 cm vs 2.32 ± 0.85 cm, *P* < 0.001).

**Conclusion:**

*GNAS* mutations are prevalent among Chinese acromegaly patients, correlating with reduced pituitary tumor sizes and enhanced GH secretion functions. Our findings underscore the influence of gender on the clinical manifestations of *GNAS* mutations. Accordingly, we recommend that future clinical and foundational research studies on acromegaly give heightened consideration to gender-specific differences.

## Introduction

Acromegaly, a rare disorder precipitated by the overproduction of growth hormone (GH), is primarily attributed to GH-secreting pituitary adenomas, accounting for more than 95% of cases (1). The precise mechanisms underpinning the formation of these GH-secreting adenomas are yet to be comprehensively understood. Recent advances have spotlighted the role of molecular mechanisms, identifying mutations in the α-subunit of stimulatory guanine nucleotide-binding protein (*GNAS*) gene as the foremost drivers of acromegaly (2, 3). These somatic, gain-of-function mutations in *GNAS* focus on codons 201 and 227 (4), resulting in constitutive activation of Gsα and increased cyclic adenosine monophosphate (cAMP) synthesis. This cascade fosters cellular proliferation and an overabundance of GH secretion (5). While somatic *GNAS* mutations are implicated in roughly 40% of pituitary GH adenomas, reported mutation rates exhibit significant variation across different ethnic groups, sample sizes and genomic analysis techniques. Some studies have investigated the association between clinical characteristics and somatic *GNAS* mutations. Typically, patients bearing *GNAS* mutations present with smaller tumor sizes, higher GH and insulin-like growth factor 1 (IGF-1) levels and better responses to neurosurgeries (4, 6, 7). However, some studies did not reach significant conclusions mentioned above (8). Furthermore, previous studies have often ignored the impact of gender on the clinical features of acromegaly. For example, it suggests that female patients with acromegaly have higher GH levels and are more susceptible to metabolic syndrome compared to males (9, 10). Therefore, our study is designed to explore the *GNAS* mutation status and gender-specific characteristics based on a large Chinese acromegaly cohort.

## Materials and methods

### Subjects and clinical data collection

Based on the clinical biobank of Peking Union Medical College Hospital (PUMCH), a total of 228 acromegaly patients were registered between May 2015 and January 2022. We excluded patients who had received any treatment before presenting to PUMCH and randomly selected 100 patients for study inclusion. Finally, 97 frozen pituitary adenoma tissues of them were successfully obtained in the biobank. All patients who registered in the biobank signed informed consent, and this study was approved by the Ethics Committee of PUMCH (ZS-0183).

We collected clinical data from the 97 patients, including their demographic features, clinical symptoms, hormonal levels, imaging examinations, treatment outcomes and follow-ups based on the electronic medical records system at PUMCH. According to the maximum diameters observed on sellar magnetic resonance imaging (MRI), GH-secreting adenomas were classified into microadenomas (<10 mm), macroadenomas (≥10 mm) and giant adenomas (≥40 mm). Tumor volume was calculated by 0.5 × width × length × height (11). A paradoxical GH increase during oral glucose tolerance test (OGTT) was defined as a peak-to-basal GH ratio ≥120% within the first 120 min of OGTT (12). Remission criteria were (1) serum fasting or random GH levels decreased to <1.0 μg/L (or nadir GH levels <1.0 μg/L in OGTT) and (2) IGF-1 decreased into the normal range matched with age and sex (13).

### Hormonal assay

Serum GH, IGF-1 and prolactin concentrations were measured by chemiluminescent immunoassay (Siemens, Germany). Blood glucose, fasting insulin, total cholesterol, triglycerides, high-density lipoprotein cholesterol and low-density lipoprotein cholesterol concentrations were measured by chemiluminescence immunoassay (Beckman, USA). The age- and sex-matched IGF-1 standard deviation score (IGF-1 SDS) was computed according to Chinese IGF-1 reference ranges reported in 2017 (14). During the 75 g OGTT, both serum GH and blood glucose levels were measured at baseline and at 30, 60, 90 and 120 minutes after oral glucose administration.

### Detection of *GNAS* gene mutation

Genomic DNA was extracted using Omega Tissue DNA Kit (Omega Bio-Tek, USA) from frozen adenoma tissues, which were stored at −80°C. Tumor genomic DNA was used as a template to amplify *GNAS* gene regions containing the two mutation hotspots in codons 201 and 227 by polymerase chain reactions (PCRs). A pair of primers was designed to amplify a 654-bp region encompassing both mutation hotspots (forward primer: GCTTCCTGGACAAGATCGAC and reverse primer: CTCCACAAACCTGTTGTTCCA). The PCR thermocycling conditions were as follows: initial denaturation at 94°C for 4 min, 36 cycles of denaturation at 94°C for 30 s, annealing at 59.9°C for 30 s, extension at 72°C for 30 s and a final extension process at 72°C for 4 min. Bi-directional Sanger sequencing was performed to evaluate *GNAS* gene hotspots mutation.

### Statistical analysis

IBM SPSS v.26.0 (IBM Corporation, USA) was used for statistical analysis. Continuous variables were presented as mean ± SD or median (Q25, Q75). Student’s t-test or the Mann–Whitney U test was used for the comparison between two continuous variables. Categorical variables were presented as fractions or percentages and compared by a Pearson *χ*^2^ or Fisher’s exact test. For ordinal categorical variables, the Mantel–Haenszel *χ*^2^ test was applied. *P* < 0.05 was considered statistically significant.

## Results

### Study population and determination of *GNAS* mutations

**Figure 1:**
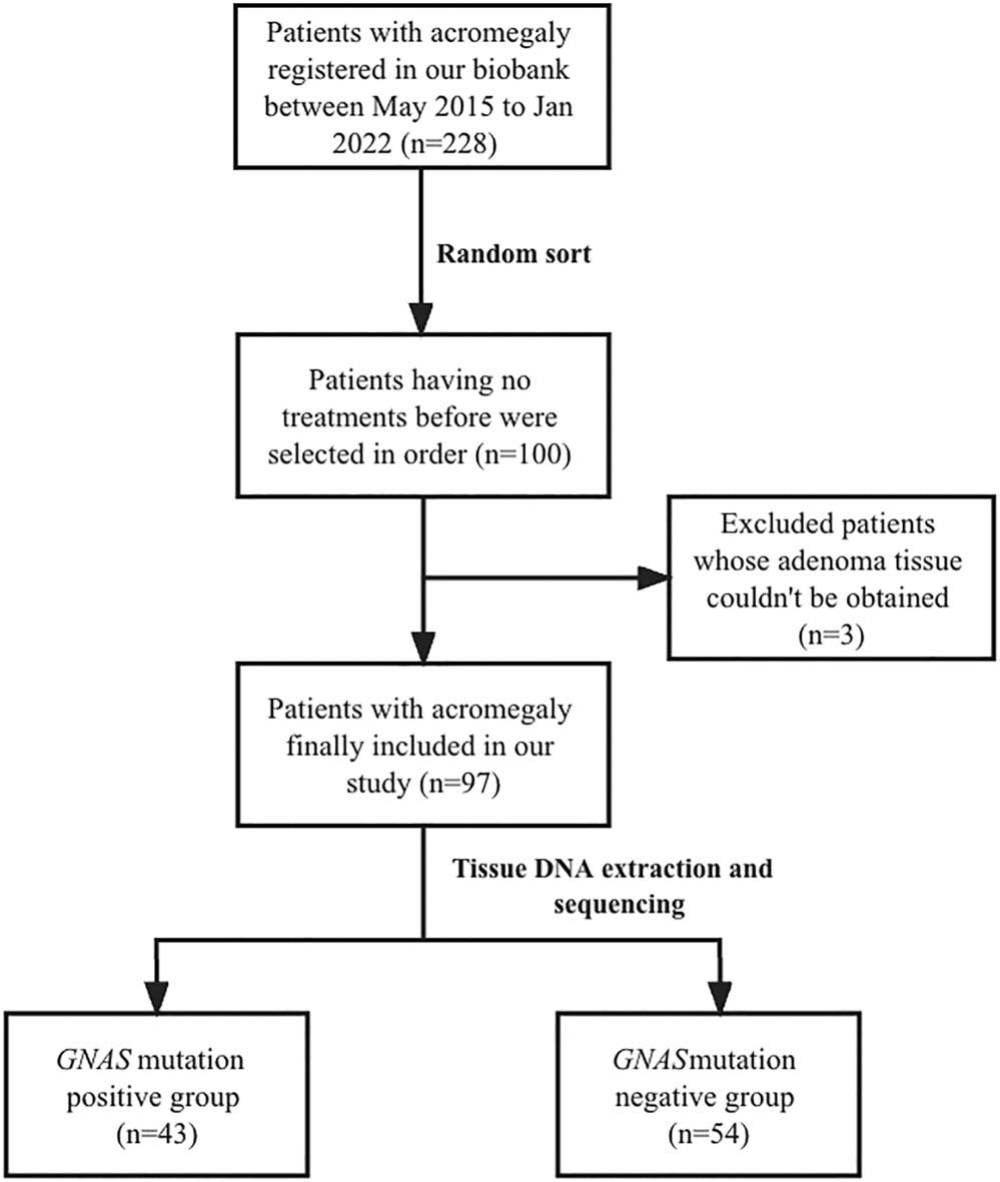
Flowchart of the study population.

Our study enrolled 97 acromegaly patients (Fig. 1). The average age of patients at the time of diagnosis was 43.3 ± 11.0 years, with males constituting 44.3% (43/97) of the cohort. The median diagnosis delay time was 60.0 (24.0, 120.0) months. Among the analyzed tumors, 9.4% (9/96) were microadenomas, 90.6% (87/96) were macroadenomas and 3.1% (3/96) were giant adenomas. The average maximal diameter of the tumors across the whole cohort was 2.02 ± 0.89 cm (0.55–5.00 cm). All patients underwent transsphenoidal surgery performed by one neurosurgical team at PUMCH, with a median post-surgical follow-up period of 7.0 (4.0, 18.0) months. At their latest follow-up, 46.7% (28/60) of the patients had achieved biochemical remission (Table 1).

**Table 1:** Clinical characteristics of acromegaly patients in relation to the presence or absence of somatic *GNAS* mutation.

	Total (*n* = 97)	*GNAS*+ group (*n* = 43)	*GNAS*− group (*n* = 54)	*P*
Gender (males)	43/97 (44.3%)	25/43 (58.1%)	18/54 (33.3%)	0.015*
Gender (females)	54/97 (55.7%)	18/43 (41.9%)	36/54 (66.7%)	0.015*
Age at diagnosis (year)	43.3 ± 11.0	43.8 ± 9.1	43.0 ± 12.4	0.726
Diagnosis delay (month)	60.0 (24.0, 120.0)	72.0 (48.0, 120.0)	36.0 (21.0, 75.0)	0.002**
BMI (kg/m^2^)	25.63 ± 3.28	26.28 ± 3.39	25.12 ± 3.13	0.082
Baseline characteristics				
Physical changes	90/97 (92.8%)	41/43 (95.3%)	49/54 (90.7%)	0.458
Headache	28/97 (28.9%)	12/43 (27.9%)	16/54 (29.6%)	0.852
Vision impairment	18/97 (18.6%)	4/43 (9.3%)	14/54 (25.9%)	0.036*
Visual field defects	7/97 (7.2%)	3/43 (7.0%)	4/54 (7.4%)	1.000
GH (ng/mL)	17.90 (9.45, 33.65)	20.80 (10.50, 35.60)	15.05 (7.43, 32.40)	0.194
GH-nadir (ng/mL)	13.65 (6.12, 26.14)	16.40 (6.54, 31.10)	12.50 (5.35, 24.20)	0.292
GH/tumor volume (ng/mL cm^−3^)	14.20 (3.89, 23.74)	18.93 (9.67, 30.12)	10.91 (2.80, 20.40)	0.005**
IGF-1 SDS	6.05 ± 1.68	6.14 ± 1.36	5.98 ± 1.90	0.630
Maximum diameter (cm)	2.02 ± 0.89	1.75 ± 0.83	2.23 ± 0.89	0.008**
Tumor volume (cm^3^)	1.74 (0.95, 5.22)	1.39 (0.54, 2.92)	3.19 (1.07, 6.13)	0.025*
Macroadenomas	87/96 (90.6%)	35/42 (83.3%)	52/54 (96.3%)	0.039*
Cavernous sinus invasion (Knosp ≥ 3)	29/85 (34.1%)	9/36 (25.0%)	20/49 (40.8%)	0.129
Impaired glucose metabolism	39/97 (40.2%)	19/43 (44.2%)	20/54 (37.0%)	0.476
Cardiac diseases	40/95 (42.1%)	21/42 (50.0%)	19/53 (35.8%)	0.165
Dyslipidemia	44/82 (53.7%)	24/37 (64.9%)	20/45 (44.4%)	0.065
Surgery				
Tumor texture soft	63/96 (65.6%)	29/43 (67.4%)	34/53 (64.2%)	0.716
Tough	26/96 (27.1%)	12/43 (27.9%)	14/53 (26.4%)	
Uneven	7/96 (7.3%)	2/43 (4.7%)	5/53 (9.4%)	
Ki-67 index 1%	29/83 (34.9%)	18/37 (48.6%)	11/46 (23.9%)	0.042*
2%	22/83 (26.5%)	9/37 (24.3%)	13/46 (28.3%)	
3–4%	26/83 (31.3%)	7/37 (18.9%)	19/46 (41.3%)	
** ≥**5%	6/83 (7.2%)	3/37 (8.1%)	3/46 (6.5%)	
AE1/AE3 (+)	67/71 (94.4%)	28/31 (90.3%)	39/40 (97.5%)	0.311
CAM5.2 (+)	61/69 (88.4%)	28/29 (96.6%)	33/40 (82.5%)	0.126
Immediate postoperative GH (ng/mL)	1.70 (0.85, 3.30)	1.70 (0.80, 4.70)	1.75 (0.98, 3.00)	0.757
Immediate postoperative IGF-1 SDS	4.85 ± 1.84	5.32 ± 1.71	4.51 ± 1.88	0.036*
Immediate postoperative GH normalization	38/97 (39.2%)	19/43 (44.2%)	19/54 (35.2%)	0.367
Follow-up				
Follow-up duration (month)	7.0 (4.0, 18.0)	4.8 (3.4, 19.0)	7.5 (4.0, 17.3)	0.466
GH at the last visit (ng/mL)	1.10 (0.48, 4.10)	0.85 (0.46, 4.80)	1.70 (0.45, 3.75)	0.706
GH-nadir at the last visit (ng/mL)	0.65 (0.17, 2.41)	0.41 (0.11, 1.58)	0.85 (0.21, 2.62)	0.372
GH normalization	40/63 (63.5%)	17/26 (65.4%)	23/37 (62.2%)	0.794
IGF-1 SDS at the last visit	1.80 (0.81, 3.35)	2.38 ± 1.82	1.71 (0.56, 2.94)	0.185
IGF-1 normalization	34/60 (56.7%)	13/25 (52.0%)	21/35 (60.0%)	0.538
Remission rate	28/60 (46.7%)	10/25 (40.0%)	18/35 (51.4%)	0.382

Abbreviations: GH, growth hormone; IGF-1 SDS, insulin-like growth factor 1 standard deviation score. *P < 0.05, **P < 0.01.

**Figure 2:**
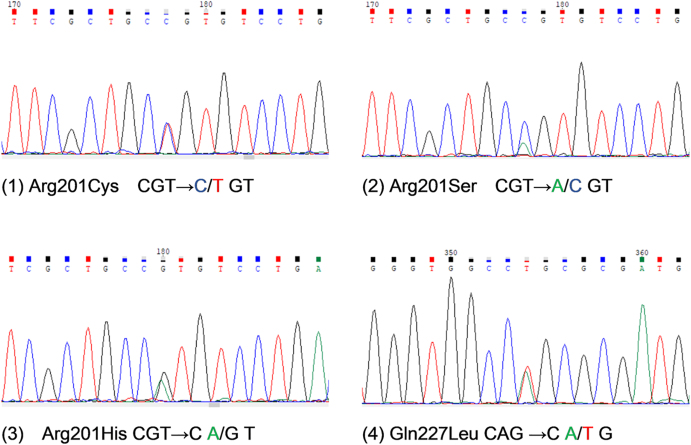
Sequence tracings for four representative *GNAS* mutations found in pituitary growth hormone adenomas.

**Figure 3:**
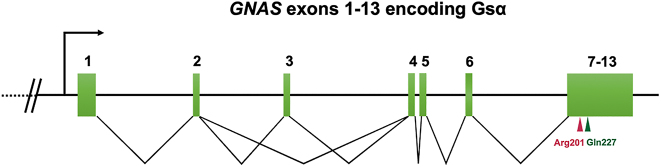
The biological structure of *GNAS* gene and location of mutant sites.

Out of the 97 patients, 43 (44.3%) carried somatic *GNAS* mutations, all of which were heterozygous missense mutations. These mutations included Arg201Cys (*n* = 33, 34.0%), Arg201His (*n* = 3, 3.1%), Arg201Ser (*n* = 2, 2.1%) and Gln227Leu (*n* = 5, 5.2%). Representative Sanger sequencing outcomes and locations of mutation sites are shown in Figs 2 and 3 (*GNAS* gene transcript: NM_000516.7).

### Comparison between patients with and without *GNAS* mutations

In terms of demographics, both groups had similar age distributions. However, patients with somatic *GNAS* mutations had a higher proportion of males (58.1 vs 33.3%, *P* = 0.015) and a longer diagnosis delay (72.0 (48.0, 120.0) vs 36.0 (21.0, 75.0) months, *P* = 0.002).

At the first visits, the symptoms of acromegaly specific physical changes, headaches and visual field defects occurred similarly in both groups. The baseline fasting GH levels and IGF-1 SDS were slightly higher in mutant patients. Notably, the GH secretion per unit volume of the tumor was significantly greater in the mutant group (18.93 (9.67, 30.12) vs 10.91 (2.80, 20.40) ng/mL cm^−3^, *P* = 0.005). According to pituitary MRI, the tumor maximum diameter (1.75 ± 0.83 vs 2.23 ± 0.89 cm, *P* = 0.008) and the proportion of macroadenomas (83.3 vs 96.3%, *P* = 0.039) were both significantly smaller in patients with *GNAS* mutations. No significant difference was found in tumor invasiveness (Knosp grade ≥ 3, 25.0 vs 40.8%, *P* = 0.129). In addition, a lower occurrence of paradoxical GH increase during OGTT was observed in the mutation group although this trend did not attain statistical significance (16.7 vs 34.0%, *P* = 0.059).

Tumor texture, assessed intraoperatively, did not show significant differences between the two groups, with the majority in both cohorts presenting soft adenomas (67.4 vs 64.2%, *P* = 0.716). According to operative records, intraoperative cerebrospinal fluid leakage occurred similarly in the two groups (20.9 vs 35.2%, *P* = 0.124). OGTT and serum GH levels were measured immediately after surgeries (within 3 days postoperatively), and there was no significant disparity in postoperative fasting GH levels (1.70 (0.80, 4.70) vs 1.75 (0.98, 3.00) ng/mL, *P* = 0.757) or OGTT-nadir GH levels (1.09 (0.37, 7.98) vs 1.10 (0.66, 2.66) ng/mL, *P* = 0.775) between the two groups. In 44.2% (19/43) of patients with *GNAS* mutations and 35.2% (19/54) of patients without *GNAS* mutations, GH levels decreased to less than 1.0 μg/L immediately after the surgery (*P* = 0.367). With regard to Ki-67 indexes, the mutant group tended to exhibit lower Ki-67 indexes (*P* = 0.042, *R* = −0.225), with only 27.0% (10/37) presenting a Ki-67 index ≥3%, compared to 47.8% (22/46) in the non-mutant group.

Among the 72 patients followed up, 26 with *GNAS* mutations and 37 without underwent no postoperative treatments. Their median follow-up durations were 4.8 (3.4, 19.0) months and 7.5 (4.0, 17.3) months, respectively (*P* = 0.466). At the last visits, the random GH levels, OGTT-nadir GH levels, IGF-1 SDS and the proportions of GH and IGF-1 SDS normalization were comparable between the two groups. Overall, 40.0% (10/25) of the mutant group and 51.4% (18/35) of the non-mutant group met the criteria for acromegaly remission (*P* = 0.382). In addition, four patients with *GNAS* mutations and five without received postoperative somatostatin receptor ligand therapy and/or radiotherapy, yet none achieved biochemical remission at their last follow-up.

### Clinical characteristics by *GNAS* mutation status across different genders

We further analyzed how *GNAS* mutations influenced the clinical characteristics among patients across different genders (Table 2). In males, patients with *GNAS* mutations exhibited significantly elevated baseline GH levels (24.40 (14.40, 36.30) vs 10.55 (5.25, 16.95) ng/mL, *P* = 0.002) and OGTT-nadir GH levels (20.00 (9.36, 32.30) vs 10.04 (4.01, 14.55) ng/mL, *P* = 0.010). However, no significant differences were observed regarding tumor volume, GH secretion per unit of tumor volume, or IGF-1 SDS between the two groups among male patients. In contrast, female patients with *GNAS* mutations had significantly smaller maximum tumor diameters (1.55 ± 0.55 vs 2.32 ± 0.85 cm, *P* < 0.001) and volumes (1.08 (0.51, 1.68) vs 3.58 (1.45, 6.07) cm^3^, *P* = 0.003) than those in the non-mutant group, which resulted in a markedly higher GH secretion per unit of tumor volume (20.83 (12.74, 29.93) vs 11.26 (2.87, 18.91) ng/mL cm^−3^, *P* = 0.013). Furthermore, female patients with *GNAS* mutations experienced longer diagnosis delay times (60.0 (42.0, 120.0) vs 36.0 (15.0, 60.0) months, *P* = 0.027) and had lower Ki-67 indexes (*P* = 0.012, *R* = −0.299). Nevertheless, baseline GH levels and IGF-1 SDS in female mutant patients did not significantly differ from those in the female non-mutant group.

**Table 2:** Clinical characteristics of acromegaly patients in relation to the status of *GNAS* gene in different genders.

	Male			Female			*P*_3_	*P*_4_
	*GNAS*+ group (*n* = 25)	*GNAS*− group (*n* = 18)	*P*_1_	*GNAS*+ group (*n* = 18)	*GNAS*− group (*n* = 36)	*P*_2_		
Age at diagnosis (year)	42.8 ± 9.1	44.4 ± 13.6	0.667	45.1 ± 9.1	42.3 ± 11.9	0.388	0.436	0.550
Diagnosis delay (month)	84.0 (54.0, 120.0)	60.0 (21.0, 120.0)	0.243	60.0 (42.0, 120.0)	36.0 (15.0, 60.0)	0.027*	0.285	0.207
Impaired glucose metabolism	11/25 (44.0%)	3/18 (16.7%)	0.059	8/18 (44.4%)	17/36 (47.2%)	0.847	0.977	0.028*
Dyslipidemia	12/21 (57.1%)	3/15 (20.0%)	0.026*	12/16 (75.0%)	17/30 (56.7%)	0.220	0.260	0.020*
Baseline GH (ng/mL)	24.40 (14.40, 36.30)	10.55 (5.25, 16.95)	0.002**	18.75 (8.00, 40.25)	17.30 (10.70, 61.73)	0.620	0.301	0.018*
Baseline GH-nadir (ng/mL)	20.00 (9.36, 32.30)	10.04 (4.01, 14.55)	0.010*	13.05 (4.99, 23.68)	14.40 (7.24, 33.53)	0.477	0.158	0.084
GH/tumor volume (ng/mL cm^−3^)	14.70 (7.41, 32.32)	10.75 (1.93, 23.14)	0.121	20.83 (12.74, 29.93)	11.26 (2.87, 18.91)	0.013*	0.513	0.811
IGF-1 SDS	6.34 ± 1.16	6.30 ± 1.67	0.925	5.86 ± 1.58	5.82 ± 2.01	0.940	0.257	0.387
Cavernous sinus invasion (Knosp ≥ 3)	4/20 (20.0%)	5/16 (31.3%)	0.470	5/16 (31.3%)	15/33 (45.5%)	0.343	0.470	0.343
Maximum diameter (cm)	1.89 ± 0.96	2.05 ± 0.96	0.612	1.55 ± 0.55	2.32 ± 0.85	<0.001**	0.185	0.282
Tumor volume (cm^3^)	1.62 (0.61, 5.10)	1.29 (0.48, 7.50)	0.961	1.08 (0.51, 1.68)	3.58 (1.45, 6.07)	0.003**	0.191	0.233
Ki-67 index								
1%	9/21 (42.9%)	4/14 (28.6%)	0.809	9/16 (56.3%)	7/32 (21.9%)	0.012*	0.370	0.709
2%	5/21 (23.8%)	5/14 (35.7%)		4/16 (25.0%)	8/32 (25.0%)			
3–4%	6/21 (28.6%)	4/14 (28.6%)		1/16 (6.3%)	15/32 (46.9%)			
≥5%	1/21 (4.8%)	1/14 (7.1%)		2/16 (12.5%)	2/32 (6.3%)			
Immediate postoperative GH (ng/mL)	1.90 (0.80, 6.10)	1.25 (0.73, 1.88)	0.175	1.25 (0.48, 3.13)	1.95 (1.13, 4.28)	0.098	0.349	0.017*
Immediate postoperative GH-nadir (ng/mL)	1.31 (0.28, 13.95)	0.88 (0.52, 1.10)	0.356	0.81 (0.37, 2.27)	1.97 (0.90, 3.02)	0.149	0.598	0.053
Follow-up duration (month)	4.8 (3.4,11.5)	9.5 (4.8, 22.9)	0.072	11.0 (3.3, 24.3)	7.0 (3.5, 13.0)	0.613	0.378	0.272
GH at the last visit (ng/mL)	0.55 (0.30, 1.43)	0.45 (0.18, 1.13)	0.381	1.60 (0.58, 7.20)	3.10 (1.40, 5.00)	0.677	0.110	0.001^**^
GH-nadir at the last visit (ng/mL)	0.19 (0.06, 1.01)	0.21 (0.15, 0.78)	0.691	0.96 (0.35, 9.18)	1.51 (0.53, 4.27)	0.868	0.077	0.010^*^
IGF-1 SDS at the last visit	2.49 ± 1.75	1.25 (0.47, 2.17)	0.060	2.23 ± 1.98	2.12 ± 2.09	0.883	0.870	0.178
Remission rate	7/14 (50.0%)	9/14 (64.3%)	0.445	3/11 (27.3%)	9/21 (42.9%)	0.465	0.414	0.214

*P*_3_: comparison between males and females in the mutant group, *P*_4_: comparison between males and females in the non-mutant group, *P < 0.05, **P < 0.01.

Abbreviations: GH, growth hormone; IGF-1 SDS, insulin-like growth factor 1 standard deviation score.

We further explored whether gender could affect the clinical features of acromegaly patients. In patients without somatic *GNAS* mutations, females displayed much higher baseline GH levels (17.30 (10.70, 61.73) vs 10.55 (5.25, 16.95) ng/mL, *P* = 0.018), a greater prevalence of abnormal glucose metabolism (47.2 vs 16.7%, *P* = 0.028) and dyslipidemia (56.7 vs 20.0%, *P* = 0.020) when compared to males. What is more, their serum GH levels were significantly higher than males immediately after surgery (1.95 (1.13, 4.28) vs 1.25 (0.73, 1.88) ng/mL, *P* = 0.017) and at their last visits (3.10 (1.40, 5.00) vs 0.45 (0.18, 1.13) ng/mL, *P* = 0.001) (Table 2). In patients with somatic *GNAS* mutations, no gender differences were identified in terms of GH levels or metabolic complications.

## Discussion

Pituitary GH-secreting adenomas appear to be monoclonal, deriving from early progenitor cells or well-differentiated pituitary cells (15), but their genetic background is still unclear. So far, several large-scale studies using next-generation sequencing methods still have not found other consistent recurrent somatic mutations like *GNAS* gene (15, 16, 17, 18, 19). As the most common driver events in GH-secreting tumors, *GNAS* mutations occur in up to 40% acromegaly patients (4), and this rate varies from 4.4% (Japanese, 1993) (20) to 59.5% (Korean, 2021) (6) in different research studies. In the present study, we found that 44.3% (43/97) of sporadic acromegaly patients carried somatic *GNAS* mutations in a large Chinese cohort. This incidence was in line with the overall mutation rate of approximately 40%, but marginally lower than the mutation rates reported in two Chinese studies from 1998 and 2016, which were 55.0% (22/40) and 54.3% (19/35), respectively (18, 21).

Our research provides an in-depth analysis of how somatic *GNAS* mutations influence the clinical features of patients with acromegaly. First, adenomas positive for *GNAS* mutations were more efficient for GH secretion, for constitutively activated Gsα could increase downstream cAMP synthesis, activate the protein kinase A (PKA) pathway and promote PIT-1 transcription and GH secretion. Second, both tumor volume and the Ki-67 index were significantly lower in adenomas with *GNAS* mutations. Previous studies have delineated the link between smaller tumor volumes and *GNAS* mutations (21, 22), but mentions of lower Ki-67 indexes are scarce. This observation could parallel discoveries in pancreatic studies, where the somatic *GNAS* R201C mutation is frequently found in low-grade precancerous lesions (23). As Kawabata and coworkers (24) pointed out, one plausible explanation could be that the cAMP–PKA pathway not only promoted tumorigenesis but also reduced tumor metastasis by inhibiting downstream NOTCH signaling. In pituitary adenomas, the dual role of cAMP in promoting DNA damage and defending against extensive DNA damage has also been documented (25). Intriguingly, a recent study introduced another viewpoint, proposing that *GNAS* mutation and *GNAS* copy number gain were mutually exclusive events in pituitary adenomas. Moreover, in patients with *GNAS* copy number gain, upregulation of cell cycle and DNA replication pathways was observed, which suggested stronger tumor cell proliferations in these patients (26).

As outlined by Lenders and coworkers (27), the effects of GH were modulated by gonadal steroids, with androgens augmenting GH activity, while estrogens diminish it by suppressing IGF-1 production in the liver. Consequently, estrogens could enhance GH secretion through feedback regulation (28). This aligns with our observation in *GNAS* non-mutant patients, where females exhibited significantly higher GH levels compared to males across all stages. We hypothesize that elevated GH levels in female patients could mask the pro-secretory impact of *GNAS* mutations seen in males. Research studies indicate that estrogen receptors can facilitate estrogen signaling, influence cAMP synthesis and engage in the PI3K and ERK signaling pathways (29, 30). Similarly, Gsα is involved in the cAMP–PKA pathway as well as the downstream PI3K and ERK pathways (31). This suggests that estrogens and *GNAS* mutations may share a common pathway, collaboratively influencing the biological characteristics of GH-secreting adenomas. However, further foundational research is essential to thoroughly examine the role of estrogen in acromegaly.

This research offers a thorough analysis of the clinical features of patients with *GNAS* mutations in a Chinese cohort, revealing pronounced secretory functions and reduced tumor dimensions in GH-secreting adenomas with *GNAS* mutations. Moreover, we first indicated that the clinical features associated with *GNAS* mutations are gender-specific, potentially related to the influence of estrogens. We proposed more attention should be paid on gender in the future research studies on GH-secreting adenomas. This study also has limitations. First, as a single-center retrospective study, the patients enrolled in our study may lack sufficient representativeness, and some clinical data, especially long-term follow-up data, are lacking. Second, more fundamental research studies are needed to investigate the relationship between estrogen and *GNAS*-related signaling pathways.

## Conclusion

In summary, we suggested that somatic *GNAS* mutations were important genetic factors in Chinese acromegaly patients. Patients with these mutations typically have smaller tumor sizes, lower Ki-67 indexes and increased GH secretion. What is more, we inventively identified gender disparities in the clinical characteristics associated with *GNAS* mutations, emphasizing the need to give importance to gender factors in future clinical and foundational research on acromegaly.

## Disclosure

A preprint has previously been published (32).

## Declaration of interest

The authors declare that there is no conflict of interest that could be perceived as prejudicing the impartiality of the work reported.

## Funding

This work was supported by the National High Level Hospital Clinical Research Funding (nos. 2022-PUMCH-A-155 and 2022-PUMCH-B-016).

## Data availability

The data used to support the findings of this study are available from the corresponding author upon request.
